# Nano helical cholesteric liquid crystals exhibit long term bistability for energy saving smart windows

**DOI:** 10.1039/d5na01103e

**Published:** 2026-02-23

**Authors:** Niveen Huseen, Ibrahim Abdulhalim

**Affiliations:** a Department of Electrooptics and Photonics Engineering, ECE School, Ilse-Katz Center for Nanoscale Science and Technology, Ben Gurion University of the Negev Beer Sheva 84105 Israel abdulhlm@bgu.ac.il

## Abstract

When an electric field is applied to a cholesteric liquid crystal (CLC) with negative dielectric anisotropy, a focal conic texture emerges depending on the helical pitch. We found that at high concentrations of chiral dopant, where the helical pitch becomes at the nanoscale (≈100 nm or less), the nano-helices comprising typically 5–10 turns can act as rigid entities with an effective dielectric anisotropy larger along the helix axis than perpendicular to it. These rigid nanohelices can reorient under an applied field between two stable configurations: a focal conic state, where the helix axis lies in the substrate plane, and a vertically aligned state. Based on this mechanism, a long term bistable smart window is demonstrated using homeotropically aligned, ultra-short-pitch CLCs. The device exhibits reversible switching between transparent and opaque, long termly stable states by tuning the amplitude and frequency of an AC electric field. Compared with longer-pitch (>500 nm) CLCs, the ultra-short helix (USH) device shows higher optical contrast, stronger scattering, and superior long-term stability. Electro-optical characterization, including Maltese cross observation, haze measurements, and contrast analysis, confirms the enhanced bistability at least up to 96 hours, with negligible degradation after a few months. This work demonstrates the superiority of USH CLCs as energy saving smart windows.

## Abstract 2

Cholesteric liquid crystals with ultrashort helical pitch are found to exhibit long-term bistability controlled by voltage and frequency. A bistable smart window is demonstrated, showing that shorter pitches yield longer bistability.

## Graphical abstract

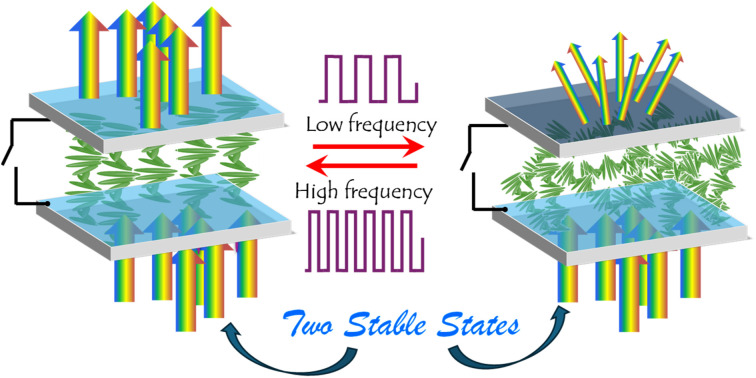

## 1. Introduction

Helicoidal nano- or micro-structured materials can form spontaneously in nature due to the chirality of their building blocks. An excellent example is chiral liquid crystals, which appear in different phases: cholesteric liquid crystals (CLCs), blue phases, and chiral smectic LCs.^1^ CLCs can also form by doping a nematic LC with chiral dopants, resulting in a helical structure with a pitch *P* inversely proportional to the dopant concentration [c] and its helical twisting power HTP following the relation: *P* = 1/[c]. HTP. This gives the freedom to design and build CLC devices with helical periods ranging from a few hundred nm, useful for visible light modulation, up to the micron scale for operation in the infrared.^2^ The tunable reflectivity window can be adjusted with voltage or temperature, providing the option to develop tunable filters and smart windows based on CLCs.^3^ CLCs are also of interest, not only in the planar alignment (PA) geometry with the helix axis normal to the substrate's plane, but also when aligned so that the helix axis is in the substrate's plane, which is called a uniformly laying helix (ULH) because they behave as a tunable diffraction grating^4^ when the period *P* is larger than the wavelength inside the medium *λ* (*P* ≥ *λ*) or as a uniform uniaxial waveplate when *P* ≪ *λ*, tunable with voltage due to the flexo-electrooptic coupling, providing a fast and linear electrooptic effect.^5,6^ The switching between the PA and ULH geometries can be achieved by changing the frequency, as at low frequencies, the electrohydrodynamic instability (EHDI) takes place and the ULH or focal conic states appear, while at high frequencies, the ions do not follow the field variations, and the LC prefers the PA orientation under high enough voltage.^7,8^

A fundamental question often raised concerns the feasibility of forming ultrashort-pitch CLCs with a helical period of 100 nm or less. Achieving such short pitches requires chiral dopants with very high twisting power incorporated at high concentrations. This leads to the next challenge: how to achieve uniform alignment of these materials, as the tightly wound helix tends to fragment into small nano-helices. Recent work by Smekhova *et al.*,^9^ showed, using resonant soft X-ray scattering, that such material is possible in a free suspended geometry (not between two substrates), forming helices of ∼104 nm pitch, and each nano-helix consists of five periods. In this work, we advance one step further by fabricating a CLC thin film with an ultrashort helical pitch of 100 nm, confined between two substrates. The as-prepared film exhibits uniform, randomly oriented domains with lateral dimensions of 100–200 µm, wherein the helical axes are oriented parallel to the substrate plane. Being composed of large domains, this state is transparent to visible light. However, by applying voltage, the domains break into the focal conic texture due to the existence of ions as a result of the EHDI, thus forming a scattering state. At sufficiently high voltage and frequency, the LC molecules, which exhibit negative dielectric anisotropy, tend to align perpendicular to the field direction; however, the nanohelices are difficult to break because the molecules are tightly twisted. As a result, the nanohelices align parallel to the field direction in a state similar to the PA state, which is transparent. Hence, the device can switch between two bistable states, one of which is opaque and the other is transparent. The stability of each state is remarkable, showing much higher stability than the CLC devices with a larger period. This highlights the potential of this device for smart window (SW).

**Fig. 1:**
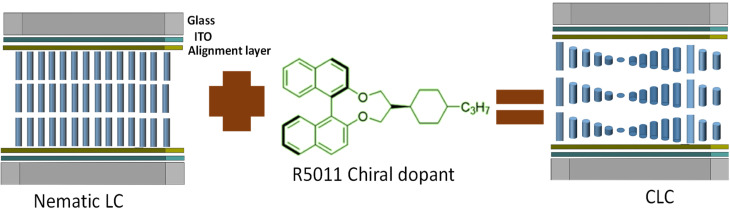
Description of the geometry of the USH-CLC device.

Traditional LC smart windows (SWs) require continuous power to maintain their optical state, leading to energy loss. Bistable SWs have evolved as an energy-efficient solution for dynamic light control in architectural and optical applications. By maintaining two stable states (transparent or opaque) without a continuous power supply, the energy consumption is reduced substantially. One of the most studied bistable systems is based on CLCs with vertically aligned helix; these systems exhibit two stable textures: the planar texture (transparent state) and the focal conic texture (opaque or scattering state).^10–12^ Beyond CLCs, smectic A (SmA) liquid crystals have also been investigated for bistable electro-optic applications.^13–16^ In contrast to the helical twist structure of CLCs, SmA phases exhibit a layered arrangement, in which the long molecular axes are oriented perpendicular to the layers. However, compared to CLC-based systems, SmA materials face certain challenges. Notably, SmA LCs require high driving voltages, which is a crucial disadvantage in the practical applications of scattering mode LC light shutters based on SmA. Additionally, while SmA systems can achieve moderate optical contrast, CLCs typically offer higher contrast between their transparent (planar) and scattering (focal conic) states. This is primarily due to the tunable helical pitch in CLCs, which allows for more effective modulation of light scattering and selective reflection.^13–16^ The bistability in such systems is governed by the balance between elastic deformation and surface anchoring energies. Bistable CLC devices focus on polymer stabilization,^10,11^ dual-frequency driving,^12^ and ion-doping for improved bistability.^11^ A recent contribution by Selvaraj *et al.*^13^ introduced a novel approach using a smectic A phase ionic liquid crystal (ILC) doped with R1011 chiral dopant. This ILC spontaneously adsorbs vertically onto the substrate without the need for alignment layers, facilitating the formation of fingerprint (FP) textures in the CLC. The resulting ILC-doped CLC system demonstrates multistable optical states, enabling stable transitions between transparent and hazy modes by tuning the voltage and frequency of the applied electric field. Notably, imperfect FP textures correspond to a transparent state that persists even after the electric field is removed. These textures arise from the vertical adsorption of ILC molecules onto the substrate, which induces partial vertical alignment of the LC. In this study, we developed a novel bistable device based on ultra-short helix CLC (USH-CLC), in which the helix with *P* ≈ 100 nm of the CLC is confined to lie within the plane of the substrate, achieved by applying DMOAP as a homeotropic alignment layer, enabling a transparent state at zero voltage (ULH state), as described in Fig. 1. The USH-CLC device demonstrates bistable optical behavior, with transitions between transparent and hazy (scattering) states modulated by the amplitude and frequency of applied voltage. By comparing it with devices having longer pitches (500 nm and longer), the ultrashort helical pitch is shown to play a critical role in both optical contrast and longer-term bistability.

## 2. Results and discussion

### 2.1. Characterizations with POM

**Fig. 2:**
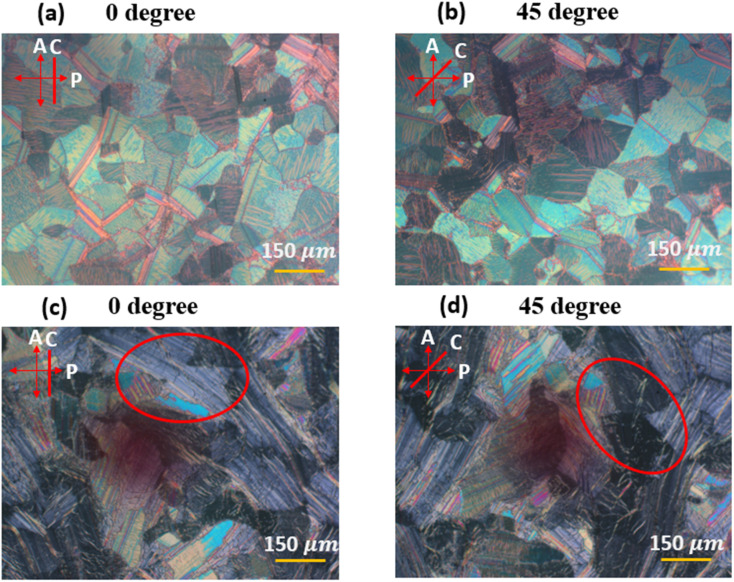
POM images of two as-prepared devices (*P* ≈ 100 nm) in the initial state (transparent state): (a) first device at 0° orientation relative to the polarizer, (b) first device at 45°, (c) second device at 0°, and (d) second device at 45°. The red ellipse highlights the same region under different orientations. *P*, *A*, and *C* indicate the transmission axes of the polarizer, analyzer, and cell long axis, respectively.

After assembly, POM images from the as-prepared devices were captured at 0 V for two different orientations of the cell (0° and 45°) relative to the polarizer. Since the devices are prepared with a vertical alignment layer, there is no preferred azimuthal orientation of the helix direction. As a result, a multidomain structure is expected. These images, taken before applying any voltage to the as-prepared device, represent the initial state of the USH-CLC structure, as shown in Fig. 2. Two devices consisting of a 10 wt% R5011 chiral dopant-LC mixture were fabricated, comparing Fig. 2a and b (first device) with Fig. 2c and d (second device), showing that the alignment quality is better in the second device. The as-prepared state exists only at the beginning, and it disappears after applying voltage. Therefore, we conclude that it is highly sensitive to the preparation conditions and to external perturbations, which can explain the difference between the two prepared devices as shown in Fig. 2. The images reveal the presence of multiple domains within the CLC layer, as expected due to the lack of an azimuthal preferred axis. At 0°, the POM images display a mixture of colored domains and extinction regions. The orientation 0° here is arbitrary and meant to be the long axis of the glass plates (*C*-axis) relative to the polarizer axis. When the cell is rotated to 45°, the colors within the domains change: extinction areas become bright and colored, while previously colored regions turn dark. This orientation-dependent optical response confirms the birefringent nature of the CLC structure and its polarization sensitivity. As the pitch of *P* ≈ 100 nm, is much less than the wavelength of visible light, the helical structure within each domain acts as a uniaxial birefringent plate.^17^

**Fig. 3:**
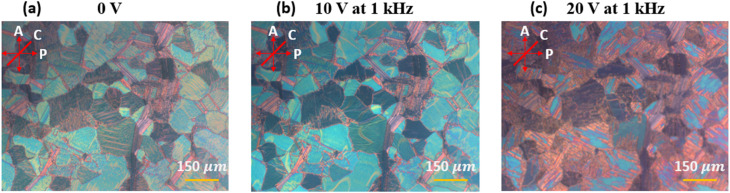
POM images of the first device in transparent state oriented at 45° relative to the analyzer, recorded under different applied voltages: (a) 0 V, (b) 10 V at 1 kHz, and (c) 20 V at 1 kHz. *P*, *A*, and *C* indicate the transmission axes of the polarizer, analyzer, and cell, respectively.

Under crossed polarizers at a 45° orientation relative to the polarizer, the device exhibits a bright, multi-colored birefringent texture at 0 V, with well-defined domains and distinct grain boundaries, corresponding to the natural helical arrangement of the liquid crystal in the absence of an electric field. When a voltage of 20 V at 1 kHz is applied, the texture becomes significantly darker and more uniform, with a notable increase in transparency. This change indicates a field-induced reorientation of the liquid crystal molecules, where the helical axis starts to orient to the PA state, showing more extinction domains as seen in Fig. 3.

**Fig. 4:**
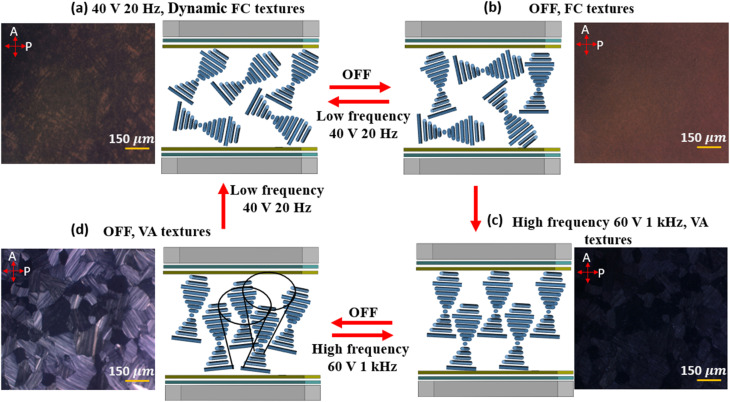
POM images and schematic illustration of the switching mechanism between modes of the USH-CLC SW, (a) FC textures (scattering state) observed under an AC field of 40 V at 20 Hz, (b) dynamic FC textures (scattering state) after the field is switched off, (c) VA textures (transparent state) under an AC field of 60 V at 1 kHz, and (d) VA textures with small domains (transparent state) after the field is removed. POM observations were performed using crossed polarizers, where *P* and *A* indicate the transmission axes of the polarizer and analyzer, respectively. The cones drawn in (d) are to illustrate that the nano helices axis may precess around the normal to the substrates, but on average, the optic axis is along the normal.

POM was also employed to investigate the scattering behavior of the USH-CLC cell under various conditions, as illustrated in Fig. 4. To aid in the interpretation of the POM images, a schematic illustration is provided. These observations were used to analyze the mechanism behind the electrically switchable dynamic light scattering mode. When a low-frequency AC electric field (40 *V*_rms_ at 20 Hz) is applied perpendicular to the substrates, the dielectric torque and ionic flow affect the negatively anisotropic CLC molecules. This induces focal conic (FC) textures, as illustrated in Fig. 4a, *via* the EHDI effect combined with dynamic light scattering, as shown in Fig. 4c. At this stage, the helical axis of the CLC becomes unstable and fluctuates due to the applied low-frequency field. After the field is removed, the LC molecules remain in a disordered FC state with small domains (Fig. 4b), influenced by surface alignment forces. Applying a high-frequency AC electric field (60 *V*_rms_ at 1 kHz) perpendicular to the substrates eliminates ionic flow effects. In this case, only the dielectric torque acts on the negative-type LC, reorienting the molecules parallel to the substrate, meaning the helix axis becomes vertical to the substrate plane, resulting in uniform textures, as shown in Fig. 4c. After the field is removed, the uniform textures persist, as shown in Fig. 4d, indicating that this state of the LC cell is transparent. The extinction between crossed polarizers in this state does not depend on the cell orientation, indicating that the average orientation of the helix is perpendicular to the substrate's plane. Since the optic axis for the nano helices is along the helix axis, an extinction will be observed between crossed polarizers, no matter what the device orientation.

### 2.2. Maltese cross observations

**Fig. 5:**
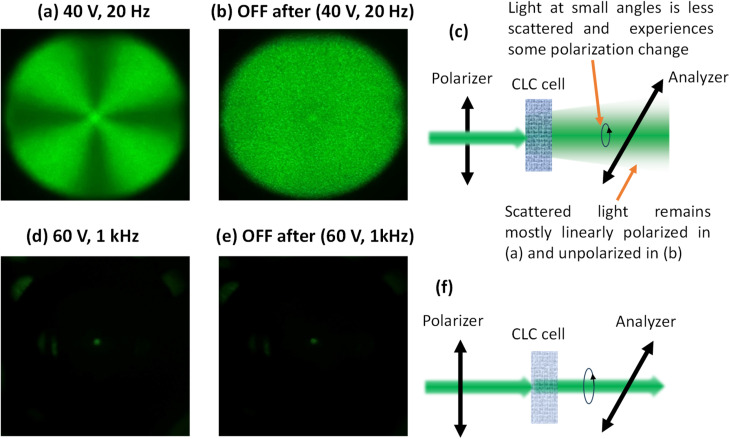
Experimental and simulated light patterns of the USH-CLC cell placed between crossed polarizers and illuminated by a monochromatic green laser (532 nm). Panels (a–c) correspond to the scattering state: (a) partially scattering under 40 V at 20 Hz, (b) almost fully scattering after switching off 40 V at 20 Hz, and (c) an illustration setup for the scattering behavior. Panels (d–f) correspond to the transparent state: (d) under 60 V at 1 kHz, (e) after switching off 60 V at 1 kHz, and (f) an illustration setup of the transparent state behavior. The light passing at zero angle (non-scattered) experiences some birefringence, thus changing its polarization to elliptical and giving rise to the central bright spot.

To further investigate the internal structure of the USH-CLC cell, we examined its response under collimated green and red laser irradiation. When an AC voltage of 40 V at 20 Hz was applied, the device entered the partially scattering state, corresponding to the FC texture. As shown in Fig. 5a, the transmitted laser pattern exhibited a characteristic dark cross (further details are included in the SI (Fig. S1 and S2)). After switching off the voltage, the device became almost totally scattering. In this case, the dark cross gradually disappeared, resulting in a smoother intensity distribution (Fig. 5b). This behavior may be attributed to further randomization of the FC domains, and their size becomes comparable to the light wavelength, thus giving larger scattering and smearing out the dark cross. Finally, when the device was switched to the vertically aligned helix texture, the transmitted light was strongly suppressed, and no laser pattern was observed on the screen, confirming the transparent state under crossed polarizers (Fig. 5d and e). This transparent state, also described in Fig. 4c and d, does not depend on the device orientation. This indicates that the helix domains are more oriented now perpendicular to the glass substrates, perhaps with a small tilt, as will be explained below. This means the average optic axis is nearly along the light propagation direction, explaining the extinction state at any orientation.

The Maltese cross is usually observed when a diverging beam passes through two crossed polarizers due to the angular dependence of the polarizers' extinction ratio, which shows dark at the central point. Here, in our case, a bright spot remains at the central point as seen in Fig. 5, indicating some effect of the liquid crystal texture at small angles. In Fig. 5a, the texture is slightly scattering, so the incident collimated beam becomes diverging, and the Maltese cross appears. The texture still has some effective birefringence, which causes some of the light at the central spot to pass through the analyzer. Upon turning the voltage off, the texture becomes almost totally scattering (Fig. 5b), and as a result, the degree of polarization of the scattered light vanishes, resulting in a uniform light transmission through the analyzer; however, the central spot becomes weaker but persists, perhaps due to residual birefringence. Fig. 5c illustrates this behavior. Note that the central spot is slightly brighter than its surroundings, simply because the scattering is not total and the non-scattered light still experiences the remaining effective anisotropy of the LC texture. In the transparent states shown in Fig. 5d and e, the texture consists of helix domains oriented nearly parallel to the substrate's normal. A small tilt of the average optic axis (helix axis) explains the remaining bright spot at the center and also the independence on the cell orientation. Fig. 5f illustrates this behavior.

### 2.3. Pictures through the smart window

**Fig. 6:**
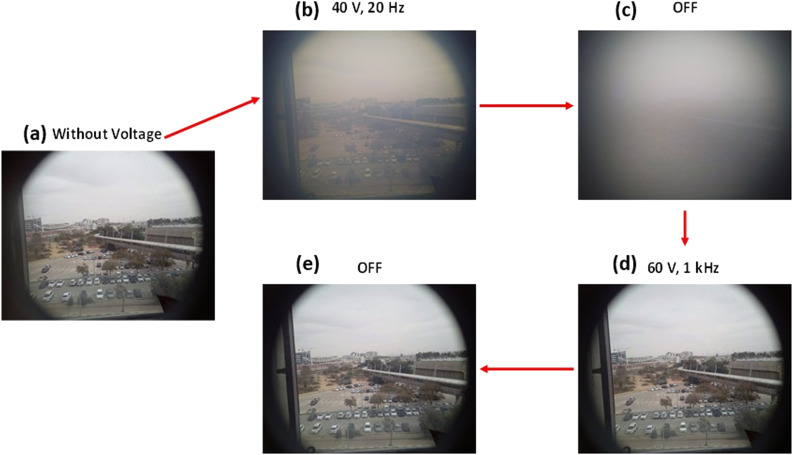
Photographs of the bistable smart window showing the transition between the two states. (a) at 0 V (transparent window), (b) under 40 V at 20 Hz (slightly scattering state), (c) after switching off the 40 V at 20 Hz (almost totally scattering state), (d) under 60 V at 1 kHz (transparent state), and (e) after switching off the 60 V at 1 kHz (transparent state). The results are for the device with *P* ≈ 100 nm if not stated otherwise.

Fig. 6 depicts the switching and bistability behavior of the HNG-7156-100 device doped with 10 wt% R5011 (2nd device with *P* ≈ 100 nm). The transparent window was achieved in the absence of voltage, as shown in Fig. 6a, then applying 40 V at a low AC frequency of 20 Hz transforms the window into a scattering state. After that, turning OFF the voltage makes it more scattering as shown in Fig. 6c. Applying a high frequency at 60 V turns the window transparent again, as shown in Fig. 6d, and turning OFF the device, the window remains transparent, as shown in Fig. 6e.

### 2.4. Scattering properties measured with an integration sphere

**Fig. 7:**
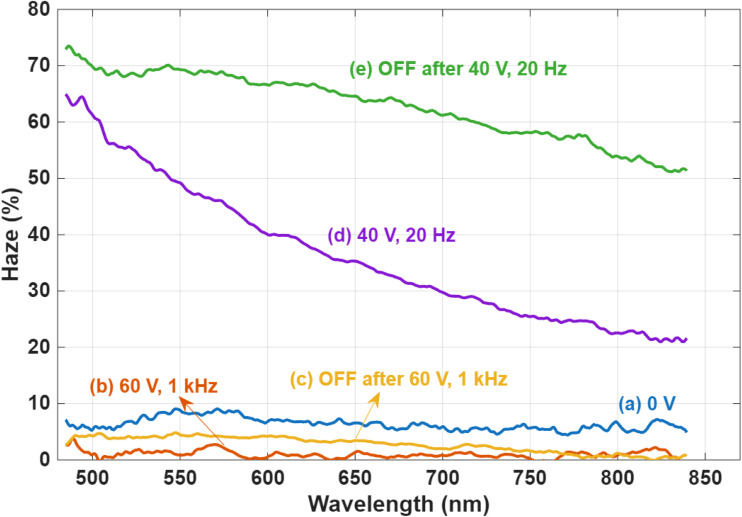
Haze measurements as a function of wavelength under different conditions: (a) at 0 V (initial state), (b) under 60 V at 1 kHz, (c) after switching off the 60 V at 1 kHz, (d) under 40 V at 20 Hz, and (e) after switching off the 40 V at 20 Hz.

As previously discussed in Fig. 6 and 7 provides a more quantitative view of the scattering behavior of the SW through haze measurements. At 0 V, the device exhibits a haze level of approximately 8%, which corresponds to the VA texture observed in the POM image of Fig. 2. This explains why the image under crossed polarizers is not completely dark; some light is still scattered by the LC texture, possibly from small domains with a size comparable to the wavelength or from the boundary between larger domains. When a high-frequency AC voltage of 60 V at 1 kHz is applied, the device transitions to a highly transparent state, resulting in a haze level close to 0%, as shown in Fig. 7b. After switching the voltage OFF, the haze slightly increases to around 4% (Fig. 7c), indicating the persistence of a nearly transparent state, consistent with bistability. Applying a low-frequency voltage of 40 V at 20 Hz drives the device into an intermediate scattering state, with the haze increasing to approximately 40% on average (Fig. 7d). Once the voltage is turned OFF, the scattering becomes even more pronounced, reaching a haze level of around 65%, as seen in Fig. 7e. This behavior is consistent with the formation of a stable FC texture, as schematically illustrated in Fig. 4. In addition, we performed the same measurement on the second device, as illustrated in SI Fig. S4, showing more or less a similar behavior, with small differences, maybe attributed to the fact that the alignment of this device looked better (higher extinction, as shown in Fig. 2) than the 1st device. The haze decays with the wavelength because the nanohelices are of size comparable to 500 nm, thus giving maximum scattering at short wavelengths and decaying for longer ones.

**Fig. 8:**
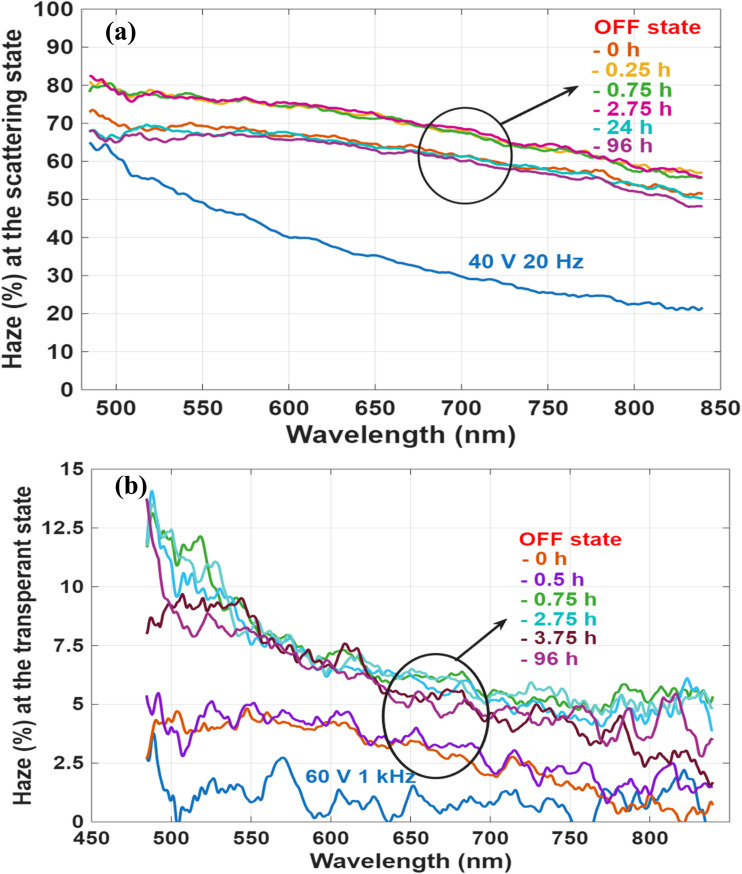
Haze as a function of wavelength at various times after switching off the LC device with 10 wt% R5011, (a) at the scattering state, (b) at the transparent state.

To evaluate the stability of the SW in both optical states, long-term haze measurements were conducted, as shown in Fig. 8. In the scattering state, the haze level remained relatively stable over time. Initially, a slight increase of approximately 10% was observed during the first few hours, likely due to the reorganization of FC domains, which temporarily enhanced the scattering. However, as the system relaxed and ionic redistribution stabilized, the haze gradually decreased, returning to a value close to that measured immediately after the voltage was turned off (at 0 h). This dynamic evolution of the FC structure is illustrated in Fig. 8a.

In the transparent state, haze measurements were also recorded over an extended period to assess the device's long-term stability. As shown in Fig. 8b, the haze level increased from an initial 5% (measured immediately after removing the voltage at 0 h) to about 15% after 96 hours. Despite this increase, the device remained visibly transparent, indicating that the stability of the transparent state is largely preserved over time, with only minor degradation in optical clarity.

**Fig. 9:**
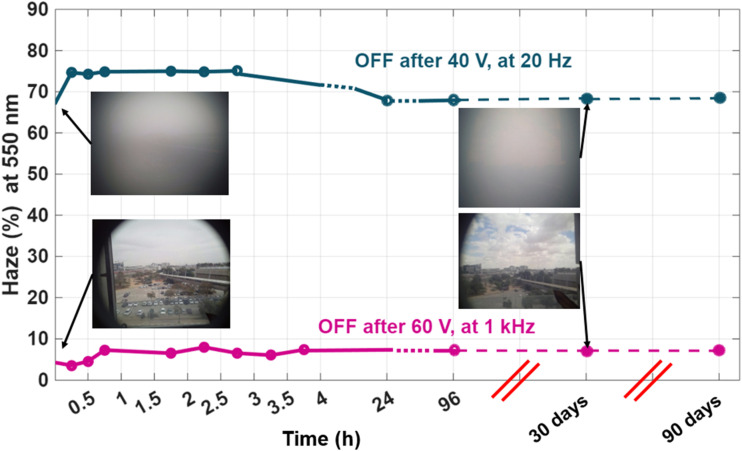
Haze *versus* time of the bistable SW based on 10 wt% R5011-LC showing little changes occurring over 96-hour period. Additionally, after 30 and 90 days the device showed the same stable states.

To further evaluate the long-term optical stability of the SW at a specific wavelength, haze measurements at 550 nm were recorded, as shown in Fig. 9. The results demonstrate that the haze levels in both the transparent and scattering states exhibit minimal variation over time, confirming the temporal stability of each optical state under ambient conditions. In addition, the full spectrum measured over 90 days has been included in the SI file as Fig. S5.

**Fig. 10:**
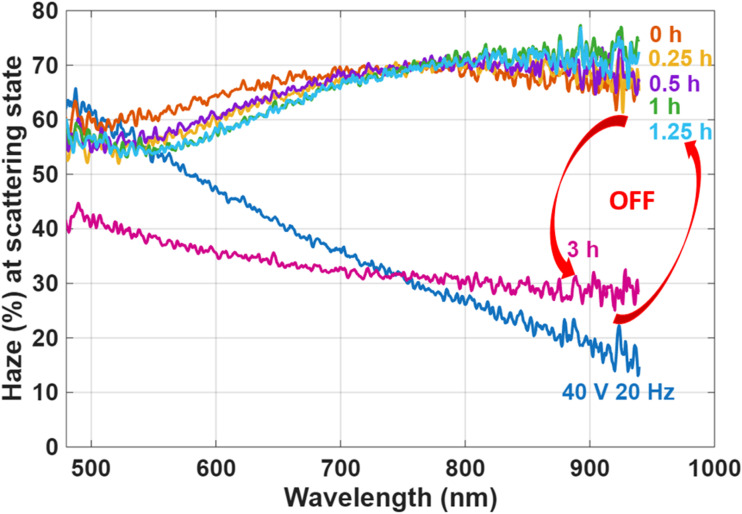
Haze as a function of wavelength in the scattering mode at various times after switching off the device with 2 wt% R5011-LC (*P* ≈ 500 nm), showing that it decays after 3 hours.

To investigate the influence of helical pitch on the bistability and long-term stability of CLC smart windows, we compare two devices that were fabricated using different concentrations of the chiral dopant R5011: 10 wt% and 2 wt%. The pitch of a CLC is found to be a critical parameter that governs its electro-optical behavior, particularly in bistable devices. It directly affects the size of focal conic (FC) domains, which in turn influences the degree of light scattering. Specifically, a shorter pitch leads to smaller domains and stronger scattering, resulting in higher optical contrast between the VA (transparent) and FC (scattering) textures. For R5011, which has a high HTP of approximately 110 µm^−1^, the devices prepared with 10 wt% and 2 wt% R5011 have estimated pitch lengths of 100 nm and 500 nm, respectively. The effect of pitch length on device performance is evident in Fig. 8 and 10, which show the haze behavior over time. The device with the longer pitch (500 nm) exhibits a noticeable degradation in the scattering state after approximately 3 hours, indicating reduced bistability. This observation aligns with findings from Cheng *et al.*,^18^ who examined the stability of various chiral dopants in CLC systems with a pitch of 1.4 µm and reported bistable scattering behavior lasting about 2 hours when using R1011. According to these observations, it seems that the bistability depends on the pitch; the lower the better the bistability. We also confirmed that a sample with 1 µm pitch made by the CB15 dopant gives only 2 hours of bistability. This is how we were led to using an ultrashort pitch of 100 nm.

In contrast, the ultra-short pitch device (100 nm) maintained its scattering state over a significantly longer duration, demonstrating enhanced bistability and temporal stability. These results highlight the importance of having ultrashort helical pitch in bistable CLC smart windows. Short-pitch structures not only enhance scattering contrast but also help stabilize the LC texture over time, making them highly suitable for applications that demand long-term retention of optical states without continuous power input.

### 2.5. Contrast measurement

**Fig. 11:**
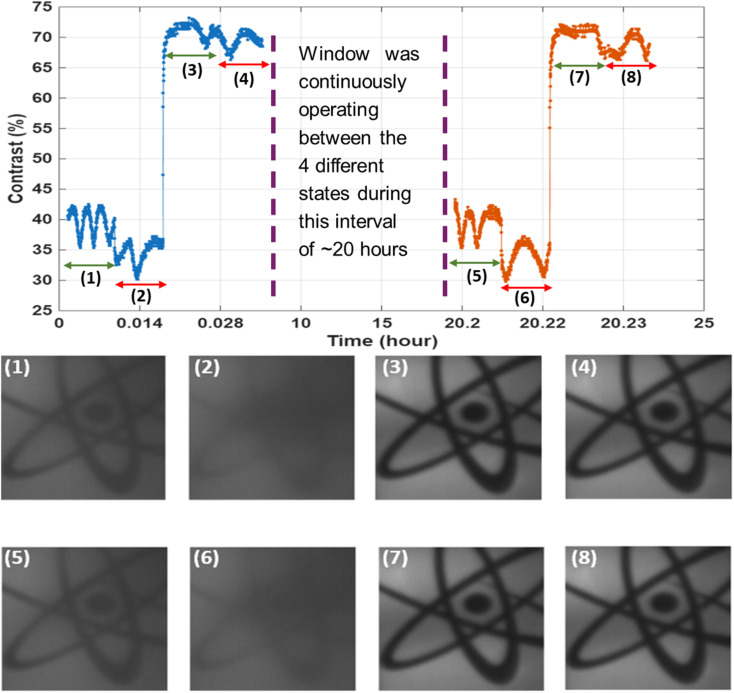
Contrast measurements were used to evaluate the stability of each mode: (1 and 5) under 40 V at 20 Hz, (2 and 6) after switching off the (40 V, 20 Hz) state, (3 and 7) under 60 V at 1 kHz, and (4 and 8) after switching off the (60 V, 1 kHz) state. The green arrow indicates when the voltage is ON, while the red arrow marks the OFF state. The results are for the device with *P* ≈ 100 nm if not stated otherwise.

**Fig. 12:**
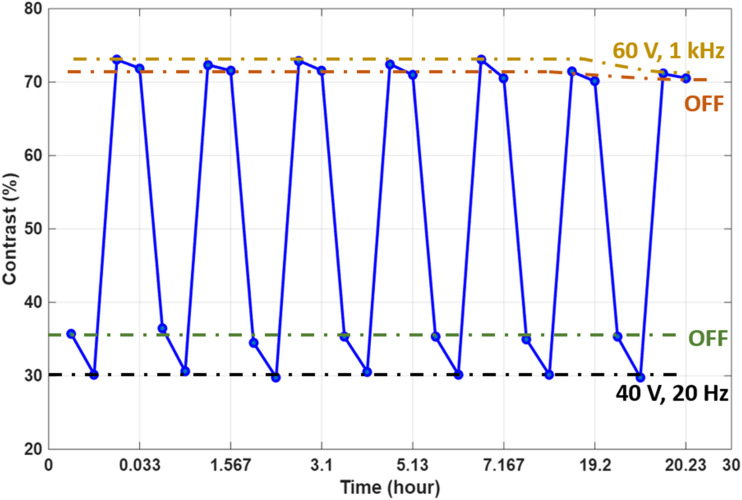
Stability for each texture as a function of time for 10 wt% R5011-LC (*P* ≈ 100 nm) device under different conditions, under 40 V at 20 Hz (black line), after switching off the (40 V, 20 Hz) state (green line), under 60 V at 1 kHz (yellow line), and after switching off the (60 V 1 kHz) state (orange line).

To evaluate the optical switching behavior and bistability of the USH-CLC smart window, the contrast was calculated using the Michelson contrast eqn (1), which is defined as,1
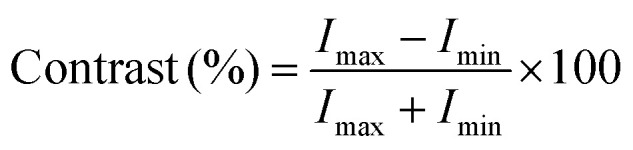
where: *I*_max_ maximum intensity (from the bright state) and *I*_min_ minimum intensity (from the dark state). The results are presented in Fig. 11 and 12. As expected from earlier observations, when the device was scattering under low-frequency, the contrast was relatively low, averaging around 37%. When the voltage was subsequently turned off, the window became even more scattering due to the stabilization of focal conic domains, and the contrast decreased further to approximately 33%, as shown in images 2 and 6 in Fig. 11.

In contrast, applying a high-frequency voltage switched the device into a transparent state, significantly increasing the contrast. In this condition, the average contrast reached approximately 70%, as shown in images 3 and 7 of Fig. 11. After the voltage was removed, the window remained in the transparent state, confirming the bistable nature of the system. The contrast under this final state remained high, at around 68%, as seen in images 4 and 8 of Fig. 11.

Fig. 12 presents the temporal stability of the smart window under different operating modes, with contrast measurements captured over time for each state. The analysis focuses on the stability of textures under four distinct conditions: low-frequency driving, post-low-frequency (OFF) state, high-frequency driving, and post-high-frequency (OFF) state. The results clearly show that for all modes, the contrast level remains nearly constant over time, indicating that the optical textures formed in each case are almost stable within the measurement period. This confirms that the device maintains strong bistability not only in the transparent state but also in the scattering state, regardless of the previously applied frequency. These findings further validate the long-term performance and reliability of the USH-CLC device.

**Fig. 13:**
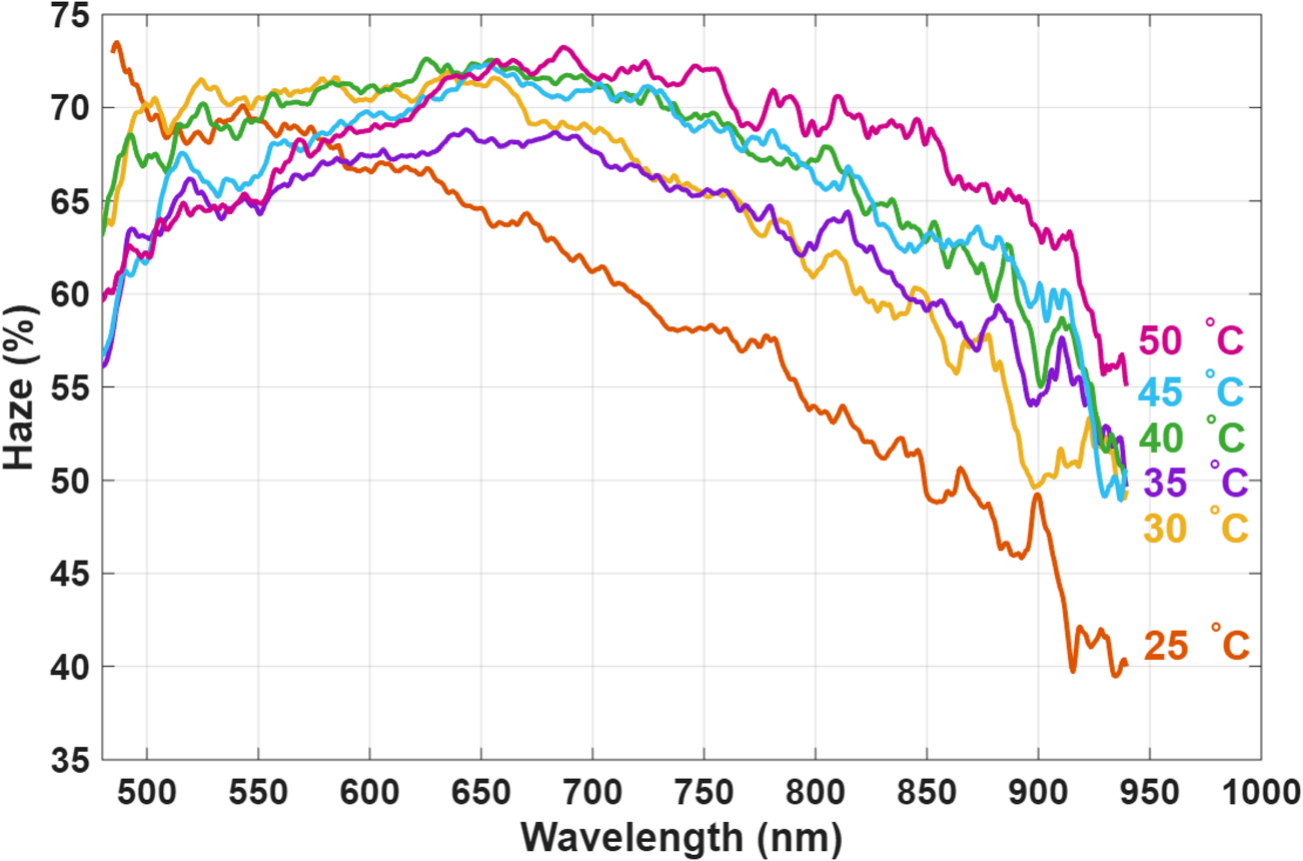
Temperature-dependent haze spectra of the bistable USH-CLC device measured under 40 V at 20 Hz.

Another characteristic of the device is shown in Fig. 13, which presents haze measurements as a function of wavelength at different temperatures measured under 40 V at 20. At 25 °C. The haze decreases at longer wavelengths because the domain size is smaller than the 700–900 nm range (nano helices are expected to be 5 periods each, hence total length of 500 nm). As the temperature increases, the domain size grows to become comparable to these wavelengths, partially because the pitch is expected to increase with temperature. Consequently, at 50 °C, the haze level rises to about 70–63%, compared to roughly 62–45% at room temperature within the same 700–900 nm range. A similar trend is observed at 30 °C and 40 °C, where the domains also increase in size, although the haze remains lower than at 50 °C.

### 2.6. Transmission measurement and response curve

**Fig. 14:**
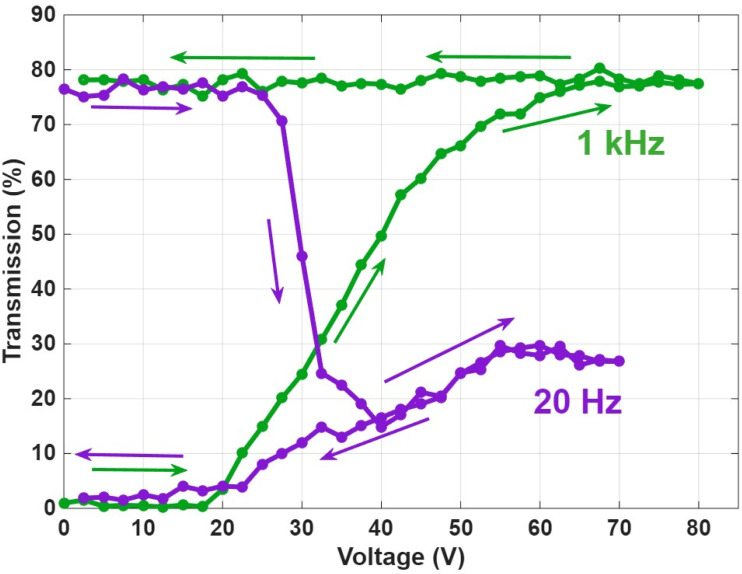
Transmittance as a function of applied voltage for the bistable PAUSH-CLC device, showing both the scattering and transparent modes.

To determine the threshold voltages required to switch the smart window between its scattering and transparent states, the transmission as a function of applied voltage was measured under both low-frequency (20 Hz) and high-frequency (1 kHz) AC conditions, as shown in Fig. 14. This analysis is crucial for identifying the optimal operating voltages that induce each bistable texture within the USH-CLC device. The results reveal that 20 Hz and 40 V for 5 seconds is sufficient to induce a stable FC (scattering) state, characterized by strong light scattering and reduced transmission. This voltage level consistently produced the scattering texture without the need for higher voltages, indicating it is the effective threshold for activating the opaque mode. Conversely, when a high-frequency AC field of 1 kHz a 60 V was required to reorient the LC molecules into the VA (transparent) state, restoring high transmission through the device. This threshold voltage ensures the LC molecules align in the plane of the substrate, overcoming ionic effects and stabilizing the transparent configuration.^20^

Based on these findings, all the switching experiments and optical characterizations presented in this study (including POM imaging, haze measurements, and contrast analysis) were conducted using these optimized voltage conditions: 40 V at 20 Hz for the scattering mode and 60 V at 1 kHz for the transparent mode. Selecting these specific voltages ensured consistent and reproducible switching behavior across all tests and validated the electro-optical bistability of the USH-CLC system.

### 2.7. Response time measurements

An additional key performance metric for electrically switchable devices is the response time, which characterizes the speed at which the device transitions between optical states. In this study, the response time was measured using a setup comprising a collimated laser diode light source, a photodetector, and a digital oscilloscope. The fall time, corresponding to the transition into the scattering state, was defined as the time required for the transmission to decrease from 90% to 10%. Conversely, the rise time, associated with the transition to the transparent state, was defined as the time taken for the transmission to increase from 10% to 90%.

**Fig. 15:**
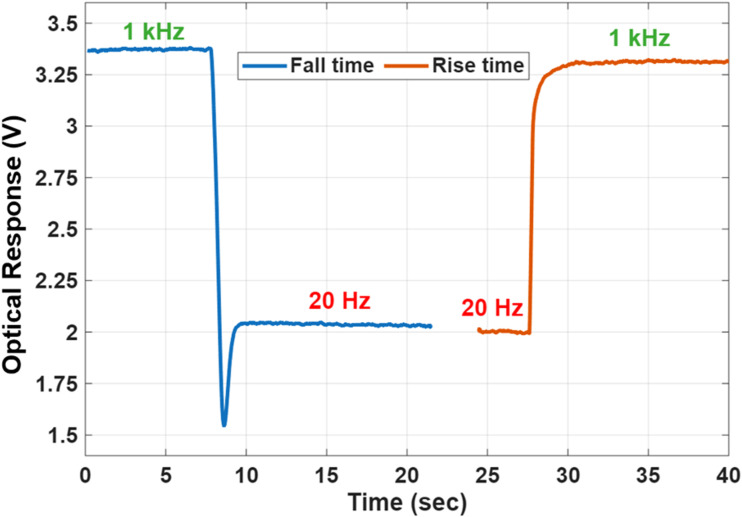
Response time at 633 nm. The blue curve shows the fall time during the frequency decrease from 1 kHz to 20 Hz, while the orange curve represents the rise time during the frequency increase from 20 Hz to 1 kHz.

As shown in Fig. 15, the measured fall time was approximately 0.94 seconds, while the rise time was significantly faster, at around 0.21 seconds. It should be noted that the analysis of response dynamics in such systems is inherently complex. It involves contributions from electrohydrodynamic instabilities (EHDI), the CLC structural response, and ionic processes such as ion generation, transport, and trapping. A detailed investigation of these interacting mechanisms and their influence on the overall response behavior is beyond the scope of this paper and is left for future work.^19^ Nevertheless, for smart windows and rewritable displays, a response time on the order of 1 s is acceptable.

As shown in Table 1, previous studies on CLCs that achieved some bistability in were predominantly based on systems with relatively long helical pitch lengths. In contrast, our work introduces a novel approach, demonstrating that ultra-short helical pitch lengths can enable long term, bistable performance (minimum 30 days). This new concept challenges the traditional assumption that longer pitches are necessary for bistability and highlights the important role of pitch-dependent domain structure in achieving long-term stability in smart window applications. In addition, fewer materials were used in this study, making it more cost-effective.

**Table 1:** Comparison table of the electro-optical characteristics of different bistable SWs (T and S refer to transmittance and scattering, respectively)

Types	Operation voltage		Materials used	Stability duration	Helical pitch	Ref.
	T to S	S to T				
PSID^a^ CLCs	20 *V*_rms_ at 50 Hz	20 *V*_rms_ at 5 kHz	CB15, 2 NLCs^d^, RM257, BME	24 hour	1.6 µm	10
Ion doped CLCs	13.1 *V*_rms_ at 50 Hz	19 V at 5 kHz	R5011, NLC, CTAB, RM257, RG184	Not measured	3 µm	11
DF-CLCs	20 *V*_rms_ at 1 kHz	20 *V*_rms_ at 100 kHz	DF-LC^c^, S811	2.5 s	1 µm	12
DFCT^b^	DC 7 V	25 *V*_rms_ at 1 kHz	QM-02-75, QM-02-77, NLC	Not measured	1.7 µm	18
Ionic LC-CLC	20 *V*_rms_ at 10 Hz	20 *V*_rms_ at 1 kHz	Ionic SmA, NLC, R1011	45 days	1.8 µm	13
ULH-CLC1	30 *V*_rms_ at 100 Hz	20 *V*_rms_ at 2 kHz	NLC and CB15	2 hour	1 µm	This work
ULH-CLC2	40 *V*_rms_ at 20 Hz	60 *V*_rms_ at 1 kHz	NLC and R5011	3 hour	0.5 µm	This work
USH-CLC	40 *V*_rms_ at 20 Hz	60 *V*_rms_ at 1 kHz	NLC and R5011	30 days	0.1 µm	This work

a PSID: polymer stabilized ion doped.

b DFCT: dynamic fingerprint chiral textures.

c DF-LC: dual frequency LC.

d NLC-nematic LC.

## 3. Conclusions

In this work, a new highly bistable smart window based on a homeotropically aligned ultrashort (∼100 nm) helical pitch CLC (USH-CLC) having negative dielectric anisotropy is demonstrated. It exhibits low operating voltage, high optical contrast, and excellent long-term stability for at least 30 days. By tuning the driving frequency, the device can be reversibly switched between its transparent and opaque states. It is proposed that due to the tight helix, the structure fragments into rigid nanohelices of a few turns each (∼5 turns), which respond to the applied electric field as if they were supramolecules with dielectric anisotropy along the helix axis larger than perpendicular to it. Importantly, both optical states remain stable even after the electric field is removed, confirming the device's bistable nature, which is essential for energy-efficient applications. Our experiments reveal that the ultra-short helical pitch (∼100 nm) plays a critical role in enhancing both scattering strength and texture stability. The short pitch leads to the formation of numerous small focal conic domains, which remain stable over extended periods, resulting in improved electro-optical performance and bistability. In contrast, devices with a longer pitch (*e.g.*, 500 nm and 1 µm) exhibited faster degradation in the scattering state and reduced optical stability with time. Additional characterization, including contrast, haze evolution, and response time analysis, further supports the effectiveness of the proposed USH-CLC device. The device demonstrated a rise time of 0.21 seconds and a fall time of 0.94 seconds, with minimal changes in optical properties over several hours. Further optical characterization under collimated green and red laser illumination provided additional insight into the internal texture. The partially scattering state exhibited a distinct Maltese cross pattern due to the controlled beam divergence at the intermediate scattering state, which gets smeared at the totally scattering state, whereas the transparent state showed nearly complete extinction, indicating well-aligned helix domains along the substrate normal. A bright spot observed at the center of the cross pattern indicates that non-scattered light experiences some polarization change due to residual birefringence. This confirms the practical feasibility of the design for smart window applications requiring low power consumption and persistent optical states. Overall, our findings suggest that optimizing the helical pitch, particularly using ultra-short pitch CLCs, is a promising strategy to develop high-performance, bistable, and energy-saving smart window devices. Being of long-term stability, the device can also be used for rewritable displays. Future work may explore material systems with even higher helical twisting power, as well as hybrid approaches to further improve switching speed and environmental stability.

## 4. Experimental section, materials and methods

### 4.1. Device preparation

First, the ITO-coated substrates were cleaned in an ultrasonic bath, followed by cleaning under an optical microscope using acetone and tissue, as described in ref. 20. Next, to achieve homeotropic alignment, the substrates were coated with a 0.1 wt% solution of dimethyloctadecyl[3-(trimethoxysilyl)propyl] ammonium chloride (DMOAP) in methanol. The coating process involved immersing the substrates in the solution for 2 minutes, followed by spin-coating to remove any excess liquid, then placing them in the oven for 30 minutes at 100 °C for drying. Afterward, mixtures of the R5011 chiral dopant at different concentrations were prepared with HNG-7156-100, a negative dielectric anisotropic liquid crystal. Finally, the liquid crystal mixture was introduced into the cell by capillary suction, with 10 µm-thick spacers used to define the cell gap.

### 4.2. Characterization methods

#### 4.2.1. POM

POM images were captured using *T* capture software under crossed polarizers to observe the texture of the USH-CLC cell. This technique enabled visualization of the focal conic and planar textures under different applied electric field conditions.

#### 4.2.2. Maltese cross observations

The Maltese cross pattern was investigated by directing a collimated laser beam through the USH-CLC cell. A screen was placed behind the sample to record the transmitted pattern. Two polarizer sheets were positioned, one before and one after the cell, to control the polarization state (as shown in SI Fig. S3). The images were captured using an IPEVO Ziggi-HD Plus Document Camera with an 8 ms exposure time and auto-lock enabled.

#### 4.2.3. Haze measurement

The haze was measured using an integrating sphere to quantify the degree of light scattering and evaluate material uniformity. Haze is a key parameter for quality control, as it helps identify internal material defects. The transmittance haze was defined as the ratio of diffuse transmittance (DT) to total transmittance (TT). Measurements followed the ASTM D1003 standard, employing an integrating sphere, a white reference standard, and a light trap.^21^

#### 4.2.4. Contrast measurement

The optical contrast was evaluated using an achromatic camera (The Imaging Source DMM-37UX226-ML) to record videos of a black-and-white target under different applied electrical conditions. The device was driven using a MATLAB-controlled AC power source, applying the following sequence of electrical signals:

• 40 V at 20 Hz (low-frequency AC field).

• Field-off state.

• 60 V at 1 kHz (high-frequency AC field).

• Field-off state again.

Videos corresponding to each optical state were recorded and analyzed using MATLAB. The contrast for each state was computed from still frames extracted from the recordings. SI videos are provided online.

#### 4.2.5. Response curve measurements

For response curve characterization, a visible light source was used in conjunction with a spectrometer to measure the transmission spectra as a function of the applied voltage at different frequencies. This setup enabled accurate evaluation of the optical response behavior by analyzing the spectral transmission changes under cyclic voltage sweeps at different frequencies.

#### 4.2.6. Response time

For the response time measurements, a laser beam was directed through the LC cell, and a photodiode detected the transmitted intensity. A function generator applied a repeating waveform to the LC cell, while both the generator and photodiode were connected to an oscilloscope. The oscilloscope displayed the applied voltage waveform and the corresponding photocurrent signal, allowing extraction of the rise and fall times from the transient optical response.

## Conflicts of interest

There are no conflicts of interest.

## Supplementary Material

NA-OLF-D5NA01103E-s001

## Data Availability

Data is available upon request from the corresponding authors. Supplementary information (SI) is available. See DOI: https://doi.org/10.1039/d5na01103e.
