# Supplementary material for: Nano helical cholesteric liquid crystals exhibit long term bistability for energy saving smart windows
Source: Nanoscale Adv. 2026 Feb 23;8(7):2257–70. doi: 10.1039/d5na01103e (PMC12936721; doi:10.1039/d5na01103e)
Supplement: NA-008-D5NA01103E-s001 [file NA-008-D5NA01103E-s001.pdf]

Supplementary Information (SI) for Nanoscale Advances.
This journal is © The Royal Society of Chemistry 2026

Nano helical cholesteric liquid crystals exhibit long term bistability for energy saving smart windows

Niveen Huseen[1], and Ibrahim Abdulhalim[1]*

[1]*Department of Electrooptics and Photonics Engineering, ECE School, Ilse-Katz Center for Nanoscale Science and Technology, Ben Gurion University of the Negev, Beer Sheva 84105, Israel.* *abdulhlm@bgu.ac.il

Figure S1 presents different orientations of the cell relative to the analyzer, while the setup used to capture these images is shown in Figure S3. It can be seen that changing the orientation of the cell does not affect the position of the dark cross.

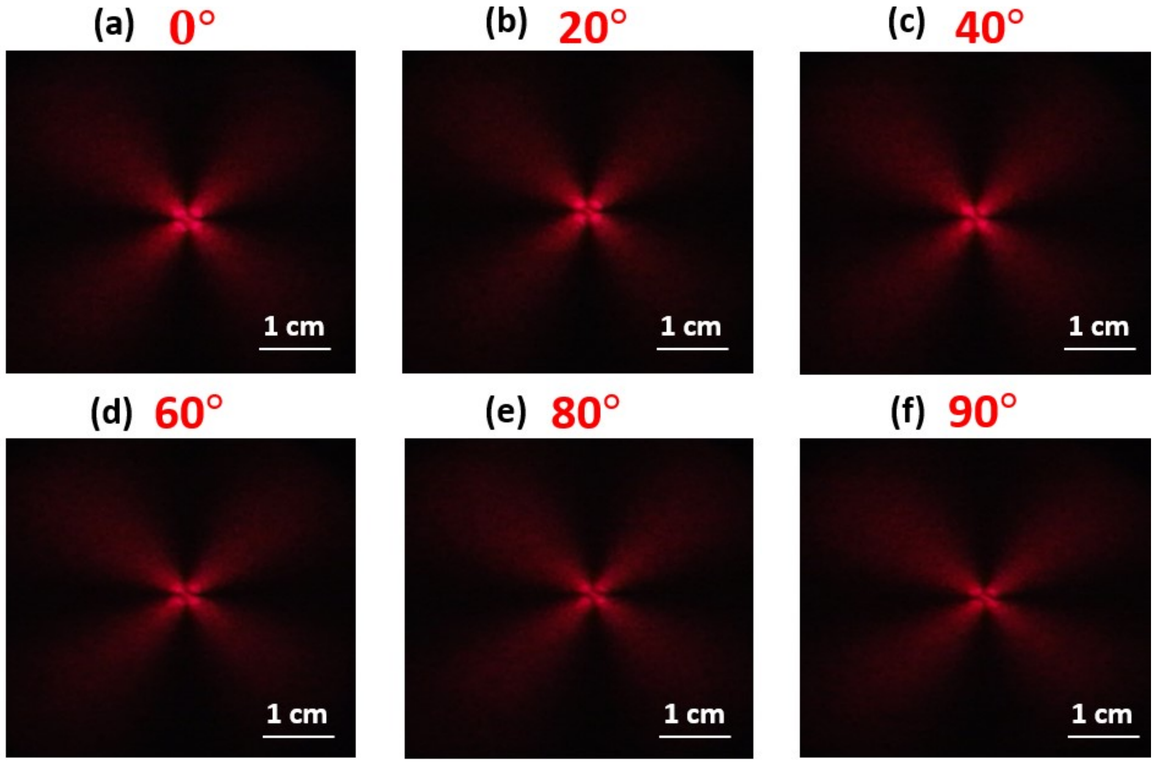

Figure S1. Light pattern obtained from the USH-CLC cell placed between crossed polarizers using a collimated He-Ne laser (633 nm) under 40 V at 20 Hz (weakly scattering state). Images show the transmitted pattern on a screen at different orientations of the cell relative to the polarizer, (a) at 0°, (b) at 20°, (c) at 40°, (d) at 60°, d) at 80°, and d) at 90°. The zero cell direction is arbitrary, in our case, along the long axis of the glass substrates.

Figure S2 shows that rotating the analyzer causes the dark cross pattern to rotate accordingly. The maximum rotation of the dark cross is 45° when the polarizer and analyzer are parallel.

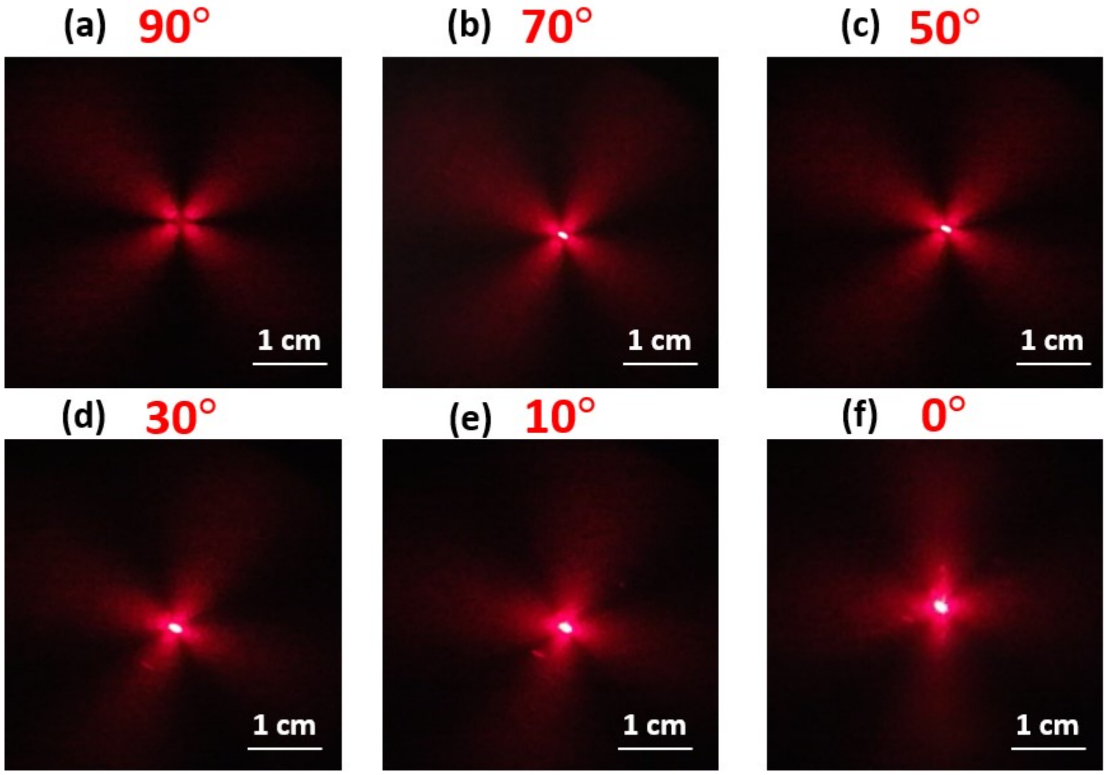

Figure S2. Similar to Figure S1, except that here the analyzer angle is changing with respect to the polarizer placed at 0° relative to the device's long axis, (a) analyzer at 90°, (b) at 70°, (c) at 50°, (d) at 30°, d) at 10°, and d) at 0°. The cross rotates by slightly less than 45° due to the remaining birefringence effect of the device in the weakly scattering state.

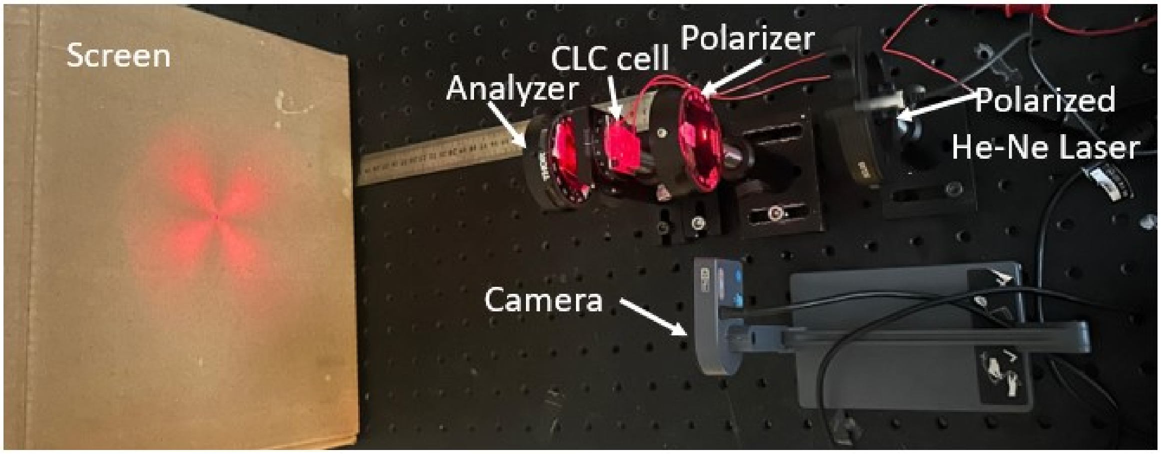

Figure S3. The setup for examining the laser pattern obtained when passing through the device between two polarizers. A collimated HeNe laser is used here, and the distance of the cell from the screen is 30 cm.

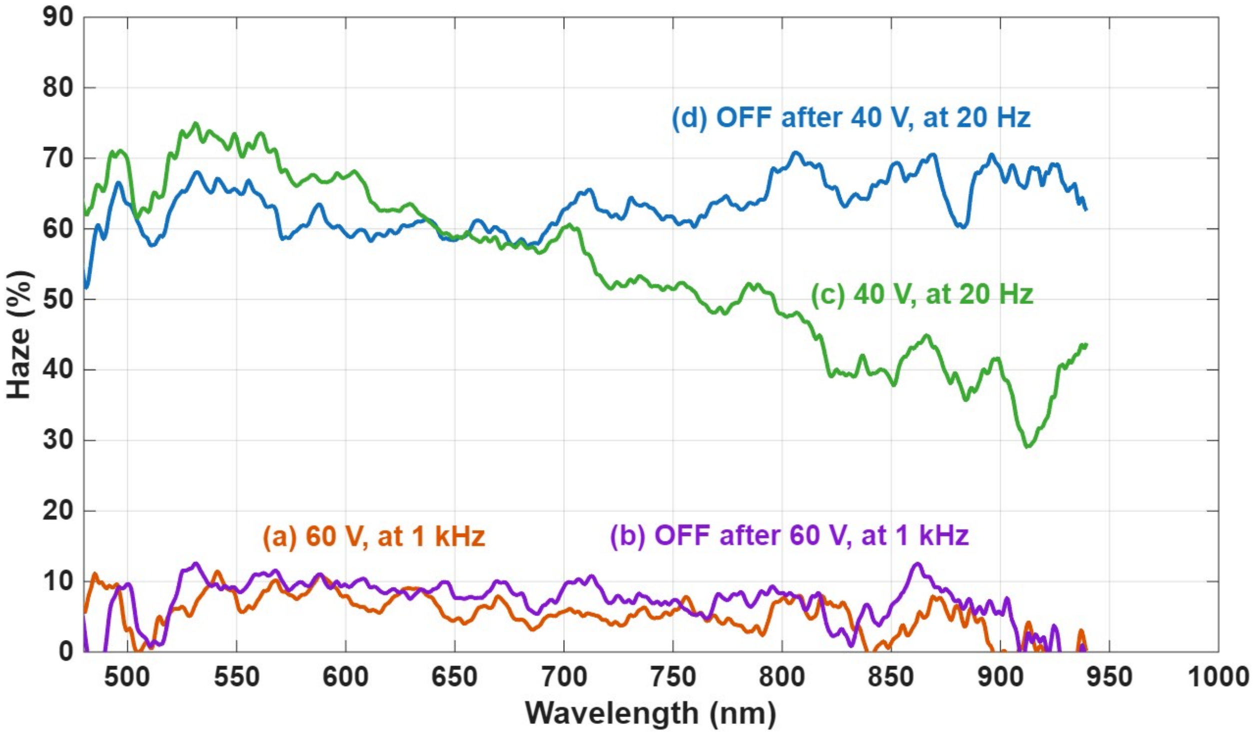

Figure S4. Haze measurements as a function of wavelength for the second device under different conditions: (a) under 60 V at 1 kHz, (b) after switching off the 60 V at 1 kHz, (c) under 40 V at 20 Hz, and (d) after switching off the 40 V at 20 Hz.

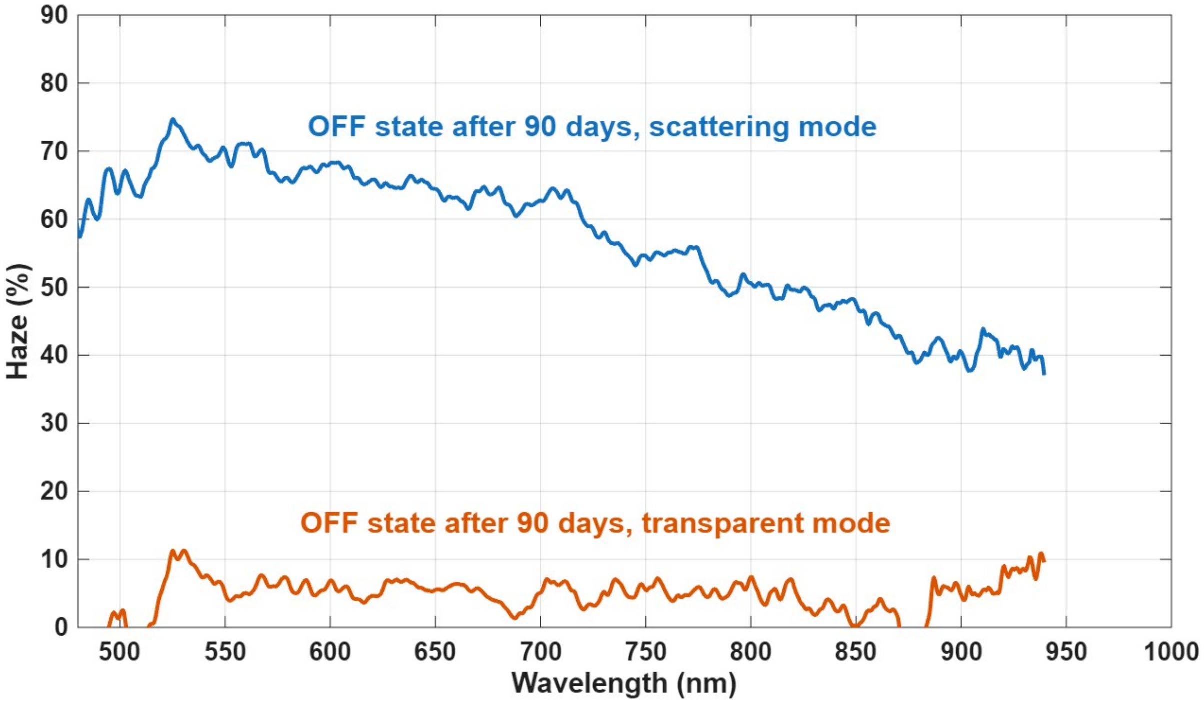

Figure S5. Haze measurements as a function of wavelength for the first device after switching off the LC device with 10 wt% R5011, a) at the scattering state, b) at the transparent state.

Videos recording to illustrate the long term bistability of the device:

First recording of the USH-CLC cell using an achromatic camera during the first operation cycle,

https://drive.google.com/file/d/1TS4vUBvfDbzu__b9X4CDfCgJmeMTZDhX/view?usp=sharing

Final recording of the USH-CLC cell using an achromatic camera during the last operation cycle after 20 hours,

https://drive.google.com/file/d/1t56j7BIElA53VSEMMVVZUarW4eHBFGxp/view?usp=sharing
